# Transcriptome sequencing to detect the potential role of long non-coding RNAs in bovine mammary gland during the dry and lactation period

**DOI:** 10.1186/s12864-018-4974-5

**Published:** 2018-08-13

**Authors:** Bing Yang, Beilei Jiao, Wei Ge, Xiaolan Zhang, Shanhe Wang, Hongbo Zhao, Xin Wang

**Affiliations:** 1 College of Animal Science and Technology, Northwest A&F University, Yangling, 712100 Shaanxi China; 2 Institute of Animal Science and Veterinary Medicine, Shandong Academy of Agricultural Sciences, Jinan, 250100 Shandong China; 3 Lab of Feed and Animal Nutrition, Tongren Polytechnic College, Tongren, 554300 Guizhou China

**Keywords:** lncRNA, Lactation cycle, Cow mammary gland, miR-221

## Abstract

**Background:**

It is known that long non-coding RNAs (lncRNAs) play an important role in various biological processes, including cell proliferation, differentiation and apoptosis. However, their functions and profiles in lactation cycle of dairy cows are largely unknown. In this study, lncRNA-seq technique was employed to compare the expression profiles of lncRNAs and mRNAs from Chinese Holstein mammary gland in dry and lactation period.

**Result:**

Totally 3746 differentially expressed lncRNAs (DELs) and 2890 differentially expressed genes (DEGs) were identified from the dry and lactation mammary glands of Holstein cows. Functional enrichment analysis on target genes of lncRNAs indicated that these genes were involved in lactation-related signaling pathways, including cell cycle, JAK-STAT, cell adhesion, and PI3K-Akt signaling pathways. Additionally, the interaction between lncRNAs and their potential miRNAs was predicted and partly verified. The result indicated that the lactation-associated miR-221 might interact with lncRNAs TCONS_00040268, TCONS_00137654, TCONS_00071659 and TCONS_00000352, which revealed that these lncRNAs might be important regulators for lactation cycle.

**Conclusion:**

This study provides a resource for lncRNA research on lactation cycle of bovine mammary gland. Besides, the interaction between lncRNAs and the specific miRNA is revealed. It expands our knowledge about lncRNA and miRNA biology as well as contributes to clarify the regulation of lactation cycle of bovine mammary gland.

**Electronic supplementary material:**

The online version of this article (10.1186/s12864-018-4974-5) contains supplementary material, which is available to authorized users.

## Background

Mammary gland is an important organ for the synthesis and secretion of milk, which provides essential nutrients for human and other animal offspring. The development and regression cycles of mammary gland include pregnancy, lactation, and involution, which is regulated by growth factors, hormones, and coding genes [1–3]. Janus kinases and signal transducers and activators of transcription ((JAK-STAT) have been shown to function downstream of several peptide hormones and cytokines that are required for postnatal development and secretory function of mammary gland [4]. In mammary epithelial cells, the phosphorylated STAT5A and STAT5B form homodimers and heterodimers to modulate differentiation, survival and proliferation through alterations in cellular gene expression [5]. The phosphatidylinositol 3-kinase-proteinkinase B/mammalian target of the rapamycin (PI3K-Akt/mTOR) signaling pathway regulates a wide range of cellular processes, such as cell proliferation, growth, survival and metastasis [6], and it is essential for mammary gland development [7]. A conditional knockout of *Akt1* averts the extended survival of mammary epithelial cells that express hyperactive *STAT5*, which indicates that the PI3K-Akt/mTOR pathway is a crucial downstream effector of JAK-STAT signaling [8].

In the past few years, many studies focused on the functions of protein-coding genes and microRNAs (miRNAs) for the development and regression cycles of mammary gland [9, 10]. *ErbB3* played a crucial role in mammary epithelial survival and differentiation during pregnancy and lactation [11]. Forty milk lipid synthesis- and secretion-associated genes, including *FADS1*, *AGPAT6*, *GPAM*, *LPIN1*, *BTN1A1*, *LPL*, *CD36*, *FABP3*, *ACSL1*, *ACSS2*, *ACACA*, *FASN*, *SCD*, *XDH*, *BDH1*, *INSIG1*, *PPARG*, and *PPARGC1A* were verified from dry period to the end of subsequent lactation period [12]. During lactation, *MAPK14*, *FRAP1*, *EIF4EBP2*, *GSK3A* and *TSC1* had an increased expression which revealed that these genes had important roles in milk protein synthesis and secretion [13]. MiRNAs are a kind of non-coding RNA, which can silence or degrade gene expression by targeting the 3’UTR region of coding gene. An increasing number of studies had demonstrated that miRNAs were involved in lactation of mammary gland by regulating their target genes [14–18]. It had been found that miR-27a could regulate cellular triacylglycerol synthesis by targeting *PPARG* gene in bovine mammary epithelial cells (BMECs) [15]. The overexpression of miR-206 changed the expression of *Wnt*, *Tbx3* and *Lef1* genes which were essential for mammary gland development, indicating that miR-206 might be a novel candidate for morphogenesis during the initiation of mammary gland formation [16]. As a downstream regulator of *PTEN*, miR-486 expressed higher during bovine high quality lactation period and could regulate the secretion of β-casein, lactose and triglyceride in BMECs, which indicated that miR-486 was required for the development of cow mammary gland [17]. In cattle, the expression of miR-221 was found to be higher in peak lactation than in early lactation, suggesting its role in the control of endothelial cell proliferation or angiogenesis [18]. In mice, miR-221 regulated lipid metabolism in mammary epithelial cells and was expressed differentially at various stages during mammary gland development [19]. MiR-212 and miR-132 were necessary for mice mammary gland development and growth by targeting *MMP9* gene, especially for mammary epithelial ducts [20].

Long non-coding RNAs (lncRNAs) are transcripts longer than 200 nucleotides, which have uncovered new layers in the control of various biological processes, including cell proliferation, differentiation and apoptosis [21]. *H19* and *SRA*, two of the earliest identified regulatory lncRNAs, may play a role in the developing mammary gland [22]. Initially, *H19* was found to function to restrict embryonic growth, but later evidence showed that *H19* had a role in long-term maintenance of adult hematopoietic stem cells [23]. Long noncoding RNA *mPINC* (mouse pregnancy-induced non-coding RNA) and *Zfas1* (Znfx1 antisense 1) had growth-suppressive roles in mammary epithelial cells [24, 25]. Previous observation demonstrated that lncRNA *Neat1* could regulate mammary gland morphogenesis and lactation by investigating the proliferation of *Neat1*-mutant cells in mice [26]. lncRNAs also could act as competing endogenous RNAs (ceRNAs) to control muscle differentiation and involve in goat lactation process [27, 28]. Pregnancy-induced noncoding RNA (*PINC*) could inhibit terminal differentiation of alveolar cells during pregnancy to prevent abundant milk production and secretion until parturition [29]. The above-mentioned studies showed that lncRNAs had crucial roles in mammary gland cell proliferation and differentiation, which would be an important issue in lactation biology. Mammary gland development and regression was directly related with cow lactation. However, the functions and profiles of lncRNAs in bovine mammary gland during dry and lactation period were largely unknown. The objective of this study was to screen the lncRNAs associated with lactation by lncRNA-seq analysis in bovine mammary gland, which would provide a new perspective for lncRNAs in lactation biology and lay the foundation for their further function study in milk composition synthesis and secretion.

## Methods

### Animals and mammary gland tissue collection

Eight healthy Chinese Holstein dairy cows at the third or fourth parities used in this study were housed in free stall and had access to water and feed ad libitum at the Experimental Farm of Northwest *A&F* University (Yangling, Shaanxi, China). After intravenous injection of lidocaine hydrochloride, approximately 4 g of mammary gland tissues were harvested via repeated biopsies from four cows at the dry period and four cows at approximately 180 days during lactation period. The tissues were dissected, frozen in RNA later (TaKaRa, Dalian, China) and stored at − 80 °C for further analysis. All experimental and surgery procedures involved in this study were approved by the Experimental Animal Manage Committee of Northwest A&F University (2011–31101684).

### Total RNA isolation and quality control for library construction

Total RNA of mammary gland tissues was isolated using Trizol reagent following the manufacturer’s instructions (Invitrogen, CA, USA). The integrity of RNA was detected using RNA Nano 6000 Assay Kit on the Bioanalyzer 2100 system (Agilent Technologies, Santa Clara, USA). RNA purity and concentration was measured using Nanodrop 2000 photometer spectrophotometer (Implen, Los Angeles, USA). The 260/280 ratio for all the samples from mammary gland tissues was about 2.0, and the RNA integrity number (RIN) was ≥8.0. After the determination of RNA purity and quality, 3.0 μg RNA per sample was used and ribosomal RNA was removed using Epicentre Ribo-zero™ rRNA Removal Kit (Epicentre, Madsion, WI, USA) for library construction. The RNA from three individuals in dry and three in lactation stage were pooled, respectively. Subsequently, the libraries were generated from the rRNA-depleted RNA pools using the NEBNext® Ultra™ Directional RNA Library Prep Kit for Illumina® (NEB, USA) following the manufacturer’s recommendations. In order to select cDNA fragments of preferentially 250~ 300 bp in length, the library fragments were purified with AMPure XP system (Beckman Coulter, Beverly, USA).

### RNA sequencing, transcriptome assembly, and quantification of gene expression level

**Fig. 1:**
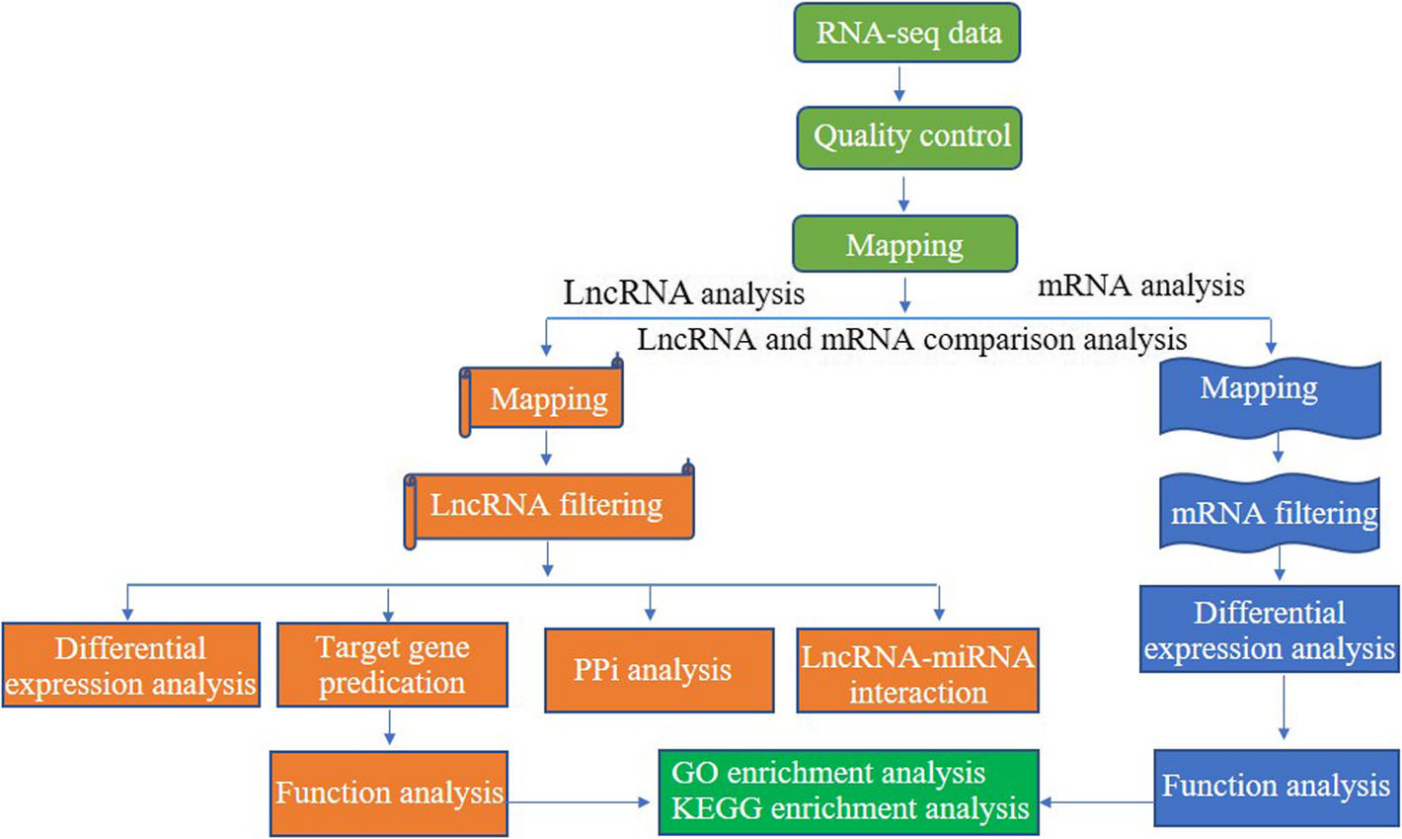
The schematic of RNA-seq analysis

The coded samples were clustered using TruSeq PE Cluster reagent (Illumina, CA, USA) according to the manufacturer’s instructions on a cBot Cluster generation system. The libraries were sequenced on an Illumina Hiseq3000 platform and 100-bp paired-end reads were generated after cluster generation. The schematic of lncRNA-seq analysis was shown in Fig. 1. Raw data were first processed using in-house Perl scripts. In this step, clean data were obtained by trimming reads containing adapter, reads containing over 10% of ploy-N, and low-quality reads (> 20% of bases whose Phred scores were < 20) from the raw data. Phred = −10log10 (e), e is defined as the error probability of sequencing for every single base. Q20, Q30 and GC content of the clean data were calculated. Subsequently, Bowtie (v2.0.6) [30] and Tophat2 [31] (v2.0.9) was used to align paired-end clean reads to the reference genome (version GCA_000003055.5_Bos_taurus_UMD_3.1.1). The default parameters for Tophat2 were set as ‘-read-mismatches=2 (≤2 mismatches are allowed) and -read-gap-length=2’ (≤2 gaps are allowed). The mapped reads were assembled using Cufflinks (v2.1.1) in a reference-based approach [32]. Cufflinks was run with ‘min-frags-per-transfrag = 0’ and ‘-library-type’, other parameters were set as default. Fragments per kb for a million reads (FPKM) of both coding genes and lncRNAs were analyzed using Cuffdiff (v2.1.1) [33]. Differential expression analysis between two groups was performed using the DESeq R package (1.8.3). The *P*-values were adjusted using the Benjamini and Hochberg method [34]. *P*-adjust (*q*-value) < 0.05 and the absolute value of fold change≥2 were set as the threshold for significantly differential expression.

### Identification the annotated and novel lncRNAs

NONCODE database was used to characterize the annotated lncRNAs in bovine from the assembled transcripts. To identify bovine novel lncRNAs, the steps were followed as Wang et al. (2017) described [35] with little modification. (1) transcripts with length < 200 bp were removed; (2) transcripts with predicted ORF > 300 were removed; (3) transcripts were compared with mRNA, rRNA, tRNA, snRNA, snoRNA and pre-miRNA (https://www.ncbi.nlm.nih.gov/) using Cuffcompare v2.1.1 to remove the same or similar transcripts [32]. (4) transcripts with FPKM < 1 were removed; (5) transcripts that did not pass the protein-coding-score test were removed using the Coding Potential Calculator (CPC) [36] and PFAM database [37].

### Prediction and functional enrichment analysis of lncRNA target genes

To reveal the potential function of lncRNAs, their target genes were predicted in *trans* and *cis*. For c*is* role, it refers to lncRNA’s action on neighboring target genes. In this study, the coding genes from 100 kb upstream and downstream of an lncRNA were searched. The *trans* role refers to the influence of lncRNAs on other genes at expression level. RNAplex bioinformatics software (http://www.bioinf.uni-leipzig.de/~htafer/index.html) was used to predict lncRNA target genes in *trans.* Genome distribution of differentially expressed lncRNAs and mRNAs was illustrated with Circos (http://circos.ca/). GO enrichment analysis was performed with DAVID database (https://david.ncifcrf.gov/). As to KEGG analysis, differentially expressed genes were analyzed with KEGG online website (http://www.genome.jp/kegg/). Protein-protein interaction network between differentially expressed genes were analyzed by STRING database (https://string-db.org/), and further visualized with Cytoscape (http://www.cytoscape.org/).

### Prediction of potential miRNAs interacted with lncRNAs

To obtain potential miRNAs interacted with lncRNAs, PicTar (https://pictar.mdc-berlin.de/), PITA (https://genie.weizmann.ac.il/pubs/mir07/mir07_prediction.html), and RNAhybrid (https://bibiserv.cebitec.uni-bielefeld.de/rnahybrid/) were used to predict the candidate miRNAs interacted with lncRNAs. The miRNAs shared by the above three tools were selected as the candidate miRNAs to assume the mechanism of lncRNAs interacted with lactation. The potential target genes of miR-221 were predicted by Targetscan (http://www.targetscan.org/vert_71/), PITA and miRanda. The genes shared by the above three tools were selected as the candidate target genes.

### Verification of sequencing data using qRT-PCR

To confirm the sequencing results, quantitative real time PCR (qRT-PCR) method was used to measure the relative expression of DEGs and DELs. The total RNAs from the cows used for lncRNA-seq were also used for qRT-PCR. The first-strand cDNA was obtained using a PrimeScript RT reagent Kit (TaKaRa, Dalian, China) following the manufacturer’s instructions. The qRT-PCR was performed in triplicate using SYBR® Premix Ex Taq™ II (TaKaRa) on the Bio-Rad CFX96 Touch™ Real Time PCR Detection System (Bio-Rad, Hercules, CA, USA). The qRT-PCR protocol was initiated with an incubation of 3 min at 95 °C, followed by 40 cycles of 95 °C for 12 s and optimized annealing temperature for 40 s. The relative expression of DEGs and DELs were analyzed using the 2^−ΔΔCt^ method and normalized by *GAPDH* gene [38]. The qRT-PCR primers for DELs and DEGs were designed with Primer Premier 6.0 (Premier, British Columbia, Canada) spanning at least one intron and shown in Additional file 1.

### BMECs transfection and expressions of four lncRNAs and ErBb3

BMECs were cultured in Dubecco’s modified Eagle medium (DMEM) /F12 containing 10% fetal bovine serum and 1% penicillin streptomycin (All from Gibco, Grand Island, NY, USA) at 37 °C with 5% CO_2_. The cells were seeded in 24-well plates, then transfected with miR-221 mimic, mimic-NC, miR-221 inhibitor and inhibitor-NC (GenePharma, Shanghai, China) at approximately 50% confluence using Lipofectamine 2000 Transfection Reagent (Invitrogen, USA) according to the manufacturer’s instructions, respectively. Briefly, Lipofectamine 2000 Transfection Reagent (1 μL) was diluted in 25 μL of Opti-MEM (Invitrogen) with miR-221 mimic, mimic-NC, inhibitor, or inhibitor-NC to yield final concentrations of 20, 20, 40, and 40 nM, respectively. The mixture was incubated at room temperature for 20 min and added to the BMECs. The transfection efficiency was assessed by fluorescence microscopy after 48 h.

Total RNA was extracted from the transfected BMECs using Trizol reagent (Invitrogen) 72 h after transfection. Then, the cDNA was obtained as above mentioned method. qRT-PCR protocol was also performed as above mentioned. Primers for the four lncRNAs and *ErBb3* are shown in Additional file 1.

## Results

### Overview of sequencing data in cow mammary gland

**Fig. 2:**
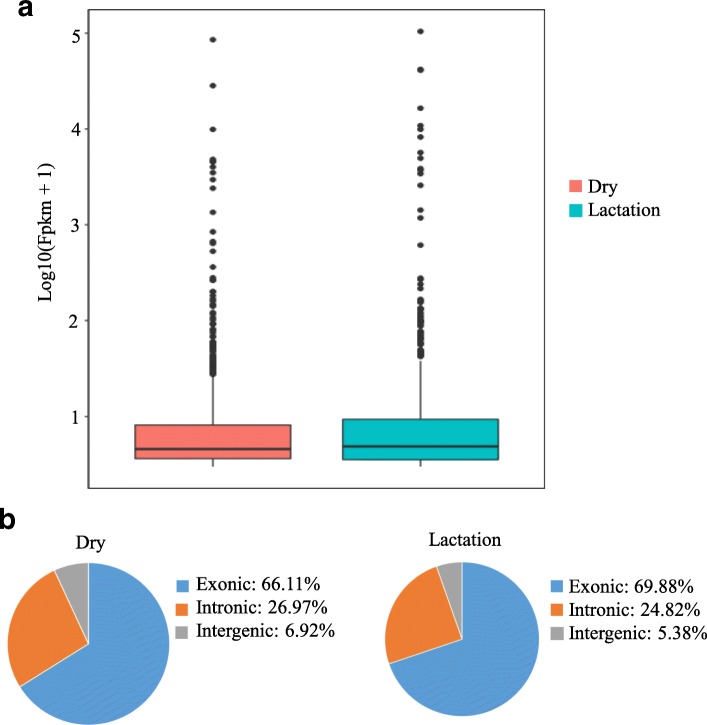
The genome distribution and relative expression of clean reads and novel transcripts. **a** The relative expression of novel transcripts and **b** the genome distribution of clean reads in cow mammary glands in dry and lactation periods, respectively

A total of 58,411,766 and 69,114,038 raw reads were produced from cow mammary glands using the Illumina Hiseq3000 platform in dry and lactation periods, respectively. After discarding adaptor sequences and low-quality sequences, 54,805,136 and 65,069,892 corresponding clean reads were obtained, and the percentage of clean reads was 93.83 and 94.15%, respectively (Table 1). The whole expression feature of transcripts was shown in Fig. 2a. The expression level of the transcripts in lactation was slightly higher than that in dry period. Similarly, 64,316 and 61,791 known transcripts, 44,651 and 43,094 known mRNAs were also obtained in dry and lactation period of cow mammary gland, respectively. Of those transcripts, 928 were novel in dry stage and 841 were in lactation, respectively.

**Table 1:** The information of sequencing data

Items	Groups	
	Dry period	Lactation period
Raw reads	58,411,766	69,114,038
Clean reads	54,805,136	65,069,892
Clean reads ratio	93. 83%	94.15%
Total transcripts	64,316	61,791
Novel transcripts	928	841
Total known mRNAs	44,651	43,094

The genome distribution of clean reads was shown in Fig. 2b. The results revealed that most clean reads in the two periods were located in exonic region, only few reads were in intergenic region. And about a quarter of the total clean reads were in the intronic region of the bovine genome.

### GO and KEGG enrichment analysis of DEGs

**Fig. 3:**
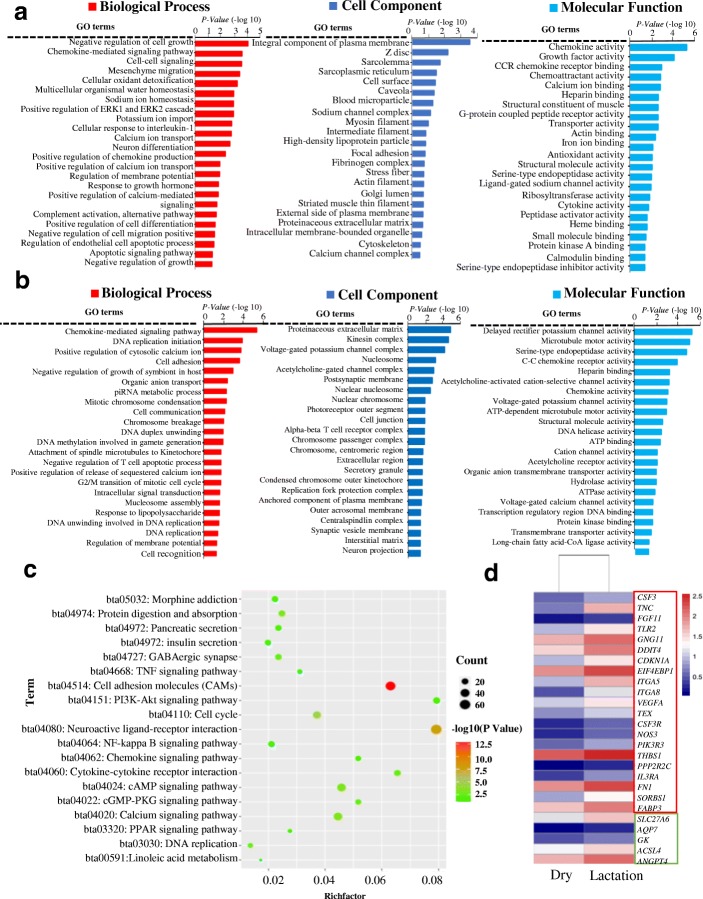
Functional enrichment analysis of significantly up- and down-regulated DEGs. **a** Gene Ontology (GO) analysis of significantly up-regulated DEGs. GO terms revealed that significantly up-regulated genes might relate to lactation-associated processes, including calcium ion binding, negative regulation of cell growth and proliferation, gene expression, etc. **b** GO analysis of down-regulated DEGs revealed that those genes might play an important role in cell division, intracellular signal transduction chemokine-mediated, and cell surface receptor signaling pathways, etc. **c** KEGG pathway enrichment analysis of significantly up- and down-regulated DEGs. KEGG terms, including cell cycle, cell adhesion molecules (CAMs), cytokine-cytokine receptor interaction, cAMP, calcium, and Wnt, PPAR, PI3K-Akt, TNF, cytokine-cytokine pathways, etc. **d** Cluster analysis of some lactation-related genes by using heat map. The genes in red box located in PI3K-Akt signaling pathway. Similarly, the genes in green box were in PPAR signaling pathway

Totally 2890 differentially expressed genes (DEGs) were obtained according the fold change≥2 and *q* < 0.01 (Additional file 2). And 2300 genes were down-regulated, whereas 590 were up-regulated in lactation compared with dry period. Then functional enrichment analysis of the 590 significantly up-regulated genes was performed based on their relative expression and fold changes. As illustrated in Fig. 3a, 69 significantly enriched GO terms (*P* < 0.05) were identified, such as negative regulation of cell growth (GO: 0030308), growth factor activity (GO: 0008083), positive regulations of ERK1 and ERK2 cascade (GO: 0070374), and sodium channel complex (GO: 0034706), etc. ERK1 and ERK2 had been suggested to play an important role in regulating cell invasion, cell proliferation, and colony formation in triple-negative breast cancer cell lines [39]. Growth factors, especially the epidermal growth factors (EGFs) and insulin-like growth factors (IGFs), were involved in development of normal mammary gland and pathogenesis of breast cancer [40].

Two thousand two hundred eight significantly down-regulated genes were also selected to carry out the functional enrichment analysis. Forty five terms, including cell division (GO: 0051301), cell adhesion (GO: 0007155), cell communication (GO: 0007154), cation transmembrane transport (GO: 0098655), and cell surface receptor signaling pathway (GO: 0035556), were shown in Fig. 3b. It was worthy to notice that the process of cell adhesion and division were closely associated with mammary gland architecture construction, maintenance, development, and lactation [41, 42].

Meanwhile, KEGG results from the significantly up- and down-regulated genes indicated that 53 significantly signaling pathways were enriched (Fig. 3c, *P* < 0.05), such as cell adhesion molecules (CAMs), PI3K-Akt, PPAR, TNF, cytokine-cytokine, cell cycle, and Wnt signaling pathways. Previous studies had determined that Wnt, PPAR, CAMs, PI3K-Akt, and TNF signaling pathways could regulate mammary gland development, milk fat synthesis, mastitis, BMECs proliferation and apoptosis [43–47]. The expression pattern of genes involved in PI3K-Akt and PPAR signaling was displayed in Fig. 3d. The heat map showed that *PIK3R3*, *CSF3*, *TNC*, *TLR2*, *GNG11*, *DOIT43*, *NOS3*, *THBS3*, *IL3RA*, *FN1*, *SORBS1*, *FAPPS*, *SLC27A*, *ACP7*, *GK*, *ACSL4* and *ANGPTL4* genes expressed higher in lactation than that in dry period at mRNA level.

### DELs and their potential interacted miRNAs involved in lactation

**Fig. 4:**
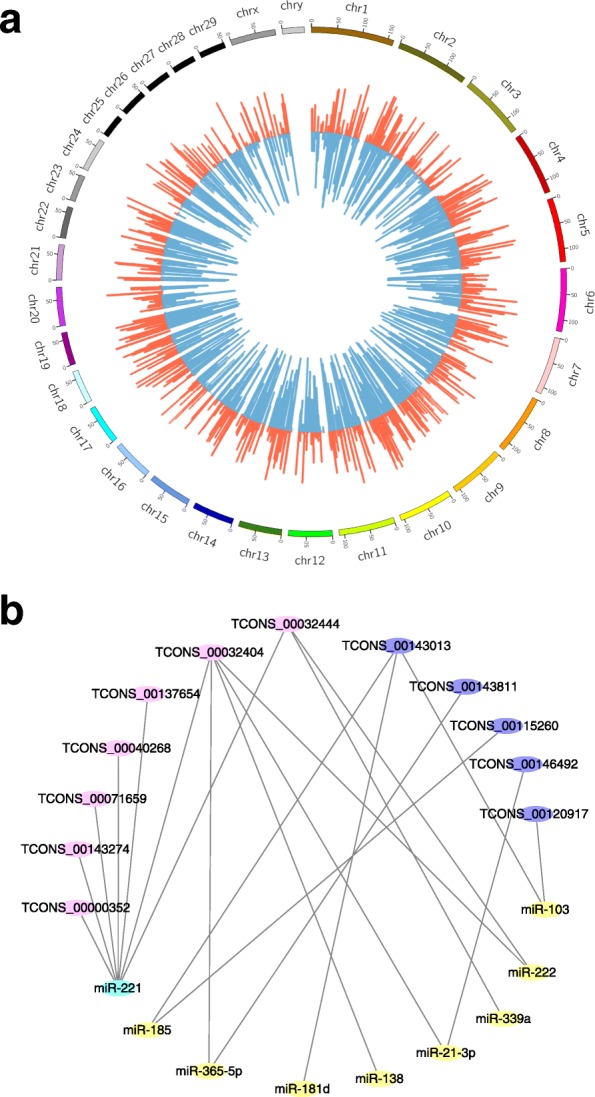
DEGs, DELs and its potential interacted miRNAs. **a** Chromosome distribution of DELs and DEGs by using Circos. Red columns represent DEGs, and blue bars represent DELs. **b** The interaction network between DELs and their potential interacted miRNAs. Those lncRNAs were depicted as pink and purple, and miRNAs as blue and yellow

Totally 23,495 expressed lncRNAs were found in the two different periods, of which 5893 were novel (Additional files 3 and 4) and 17,602 were annotated in NONCODE (Additional file 5). A total of 3746 significantly differentially expressed lncRNA transcripts were found in lactation compared with that in dry period, including 2732 down- and 1014 up-regulated lncRNA transcripts (fold change≥2, *P* < 0.05) (Additional files 6 and 7). The genome wide distribution of DELs and DEGs was shown in Fig. 4a. It could be seen that the DELs and DEGs was harmoniously located on autosomes and X chromosome. The stage-specific expressed lncRNAs and genes were shown in Additional files 8 and 9. One hundred forty five lncRNAs were identified and subjected to further analysis according their expression fold changes and genomic locations. The potential interacted miRNAs with the selected 145 lncRNAs were predicted by miRanda, PITA and RNAhybrid, and partial result was shown in Table 2. It could be seen that miR-103, miR-21-3p, miR-27a-5p, miR-107, and miR-24-3p were predicted to interact with lncRNA TCONS_00120917. The interaction network between candidate lncRNAs and their potential miRNAs was constructed (Fig. 4b). It showed that several miRNAs might interact with multiple lncRNAs, such as miR-221 interacting with lncRNAs TCONS_00032404, TCONS_00032444, TCONS_00040268, TCONS_00137654, TCONS_00071659, TCONS_00143274 and TCONS_00000352. Coincidentally, it had demonstrated that the expression of miR-221 was found to be higher in peak lactation than in early lactation, suggesting a role in the control of endothelial cell proliferation or angiogenesis which was closely related with lactation [19], so those above mentioned lncRNAs could be considered as important candidates for lactation.

**Table 2:** Differently expressed lncRNAs and their potential lactation-related miRNAs

LncRNAs	potential miRNAs interacted with lncRNAs
TCONS_00088220	miR-103, miR-21-5p, miR-138, miR-365-5p, miR-146b, miR-146a, miR-181d, miR-185
TCONS_00143013	miR-103, miR-181a, miR-30b-3p, miR-181b, miR-34a, miR-181d, miR-206
TCONS_00120917	miR-103, miR-21-3p, miR-27a-5p, miR-107, miR-24-3p
TCONS_00115260	miR-30b-3p, miR-34b, miR-34a, miR-185
TCONS_00146492	miR-21-3p, miR-17-3p, miR-24-3p, miR-34a
TCONS_00073062	miR-181b, miR-214, miR-181d
TCONS_00113482	miR-16b, miR-17-5p, miR-152
TCONS_00052219	miR-21-3p, miR-365-5p, miR-504
TCONS_00032444	miR-221, miR-222, miR-17-3p, miR-34c, miR-212
TCONS_00040268	miR-221, miR-222, miR-204, miR-211, miR-425-3p
TCONS_00137654	miR-221, miR-16a, miR-195, miR-193a
TCONS_00071659	miR-221, miR-25, miR-541, miR-370, miR-432
TCONS_00000352	miR-221, miR-151-5p, let-7b, miR-432
TCONS_00032415	miR-103, miR-15a, miR-181d
TCONS_00032449	miR-185, miR-504, miR-940

### The interaction between lncRNAs and miR-221 as well as miR-221 and ErBb3

**Fig. 5:**
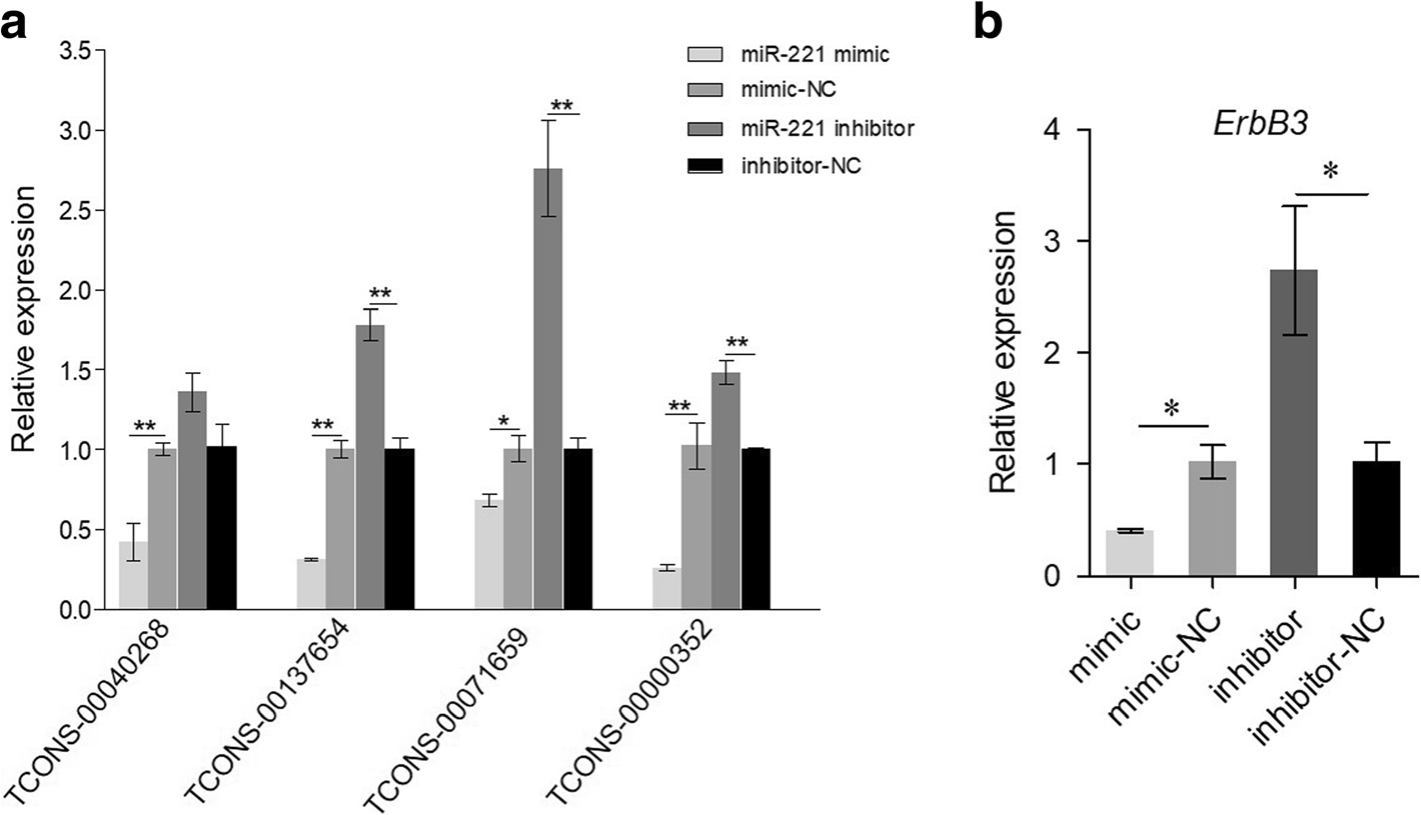
The interaction between lncRNAs and miR-221 as well as miR-221 and *ErBb3* gene. **a** The relative expression of 4 lncRNAs (TCONS_00040268, TCONS_001371654, TCONS_00071659, and TCONS_00000352) changed with the overexpression and inhibition of miR-221 **b** miR-221 could target *ErBb3* gene by transfecting miR-221 mimic and inhibitor in BMECs

To verify the interaction between lncRNAs and miR-221 and the interaction between miR-221 and *ErBb3*, overexpression and inhibition of miR-221 was employed in BMECs. Our data showed that the expressions of the four lncRNAs (TCONS_00040268, TCONS_00137654, TCONS_00071659, and TCONS_00000352) decreased with the overexpression of miR-221. On the contrary, their expressions were increased with the inhibition of miR-221 in BMECs (Fig. 5a), which indicated that miR-221 could interact with these four lncRNAs; Furthermore, the overexpression of miR-221 could reduce the expression of *ErBb3* gene in miR-221 mimic group compared with mimic-NC group. While the inhibition of miR-221 increased *ErBb3* gene expression, which indicated that *ErBb3* gene was the target of miR-221 (Fig. 5b).

### Interaction network between the candidate lncRNAs and their potential target genes

**Fig. 6:**
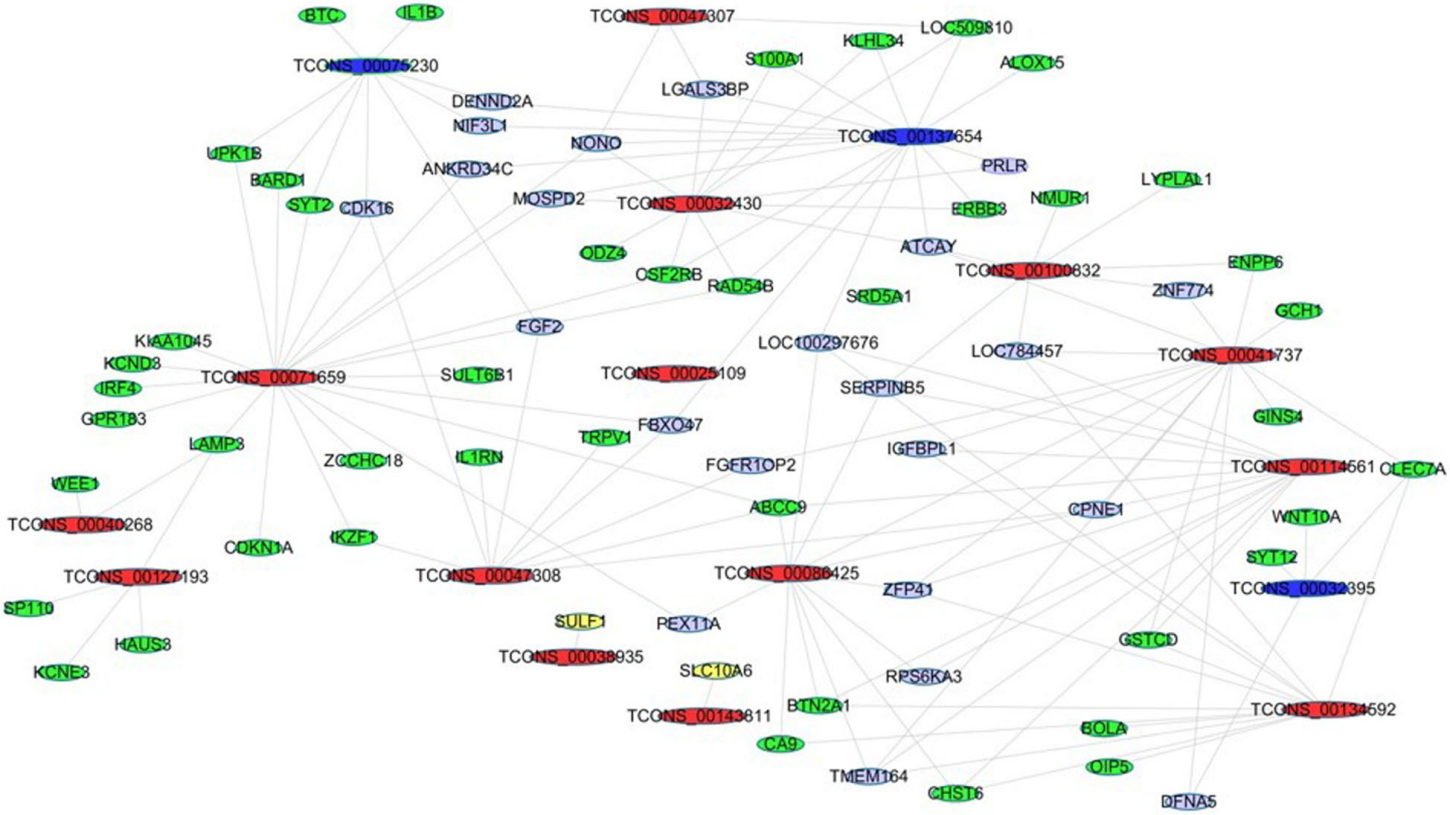
The interaction network between candidate lncRNAs and their potential target genes. The red boxes indicated up-regulated candidate lncRNAs. And the blue boxes were down-regulated lncRNAs. The up- regulated genes in the dry period were depicted as green and down-regulated genes as,yellow, respectively

To reveal the potential function of these selected lncRNAs, their target genes were predicted *in cis* and *trans* roles, and we found that the mammary gland biology related genes, such as *PRLR*, *FAS*, and *MAP3K7* genes, could be targeted by several lncRNAs *in cis* or *trans* (Table 3). In addition, the network between lncRNAs and target genes was constructed*.* The potential target genes of those lncRNAs included *S100A1*, *UPK1B*, *BARD1*, *FGF2*, *IGFBP1*, *ALOX15*, *KLH34*, *SYT12*, *WNT10A*, *SULF1*, *SLC10A6* and *CLE7A* (Fig. 6). Moreover, the expression level of those genes in dry period were significantly higher than that in lactation period in our study while the corresponding lncRNAs were significantly lower, suggesting an opposite expression trend exists between those genes and corresponding lncRNAs. Previous data had demonstrated that *IL1B* might function as a retinoic acid induced gene and could inhibit the proliferation of normal human mammary epithelial cells [48]. Therefore, it could be deduced that TCONS_00075230 might involve in regulating the proliferation of mammary epithelial cells by interacting with *IL1B* gene in cow mammary gland.

**Table 3:** DELs and their potential target genes involved in lactation

lncRNAs roles	lncRNAs	Genes
lncRNA *in trans*	TCONS_00000352, TCONS_00032430, TCONS_00137654	*PRLR*
	TCONS_00000352, TCONS_00032430, TCONS_00071659, TCONS_00137654	*SLC5A1*
	TCONS_00041737, TCONS_00047308, TCONS_00134592, TCONS_00086425	*FAS*
	TCONS_00086425, TCONS_00114561	*MAPKAPK5*
	TCONS_00114561, TCONS_00134592	*IGFBPL1*
	TCONS_00000352, TCONS_00032430, TCONS_00137654	*SLC19A3*
	TCONS_00000352, TCONS_00032430	*S100A1*
	TCONS_00041737, TCONS_00071659	*EIF2*
	TCONS_00075230	*CCNL1*
lncRNAs *in cis*	TCONS_00052219	*SLC10A7*
	NONBTAG015998.2	*ESRP1*
	TCONS_00161234, TCONS_00159090	*MAP3K7*
	TCONS_00143059, TCONS_00143062, TCONS_00143060	*PTPN13*
	NONBTAG007590.2	*STAT1*
	NONBTAT012173.2	*CCNB1*
	NONBTAT017008.2, NONBTAT017009.2	*CDK1*
	TCONS_00112212	*CD5*
	NONBTAT002501.2	*CDKN3*
	NONBTAG020902.1	*CXCL12*

### GO and KEGG analysis of lncRNAs potential target genes

**Fig. 7:**
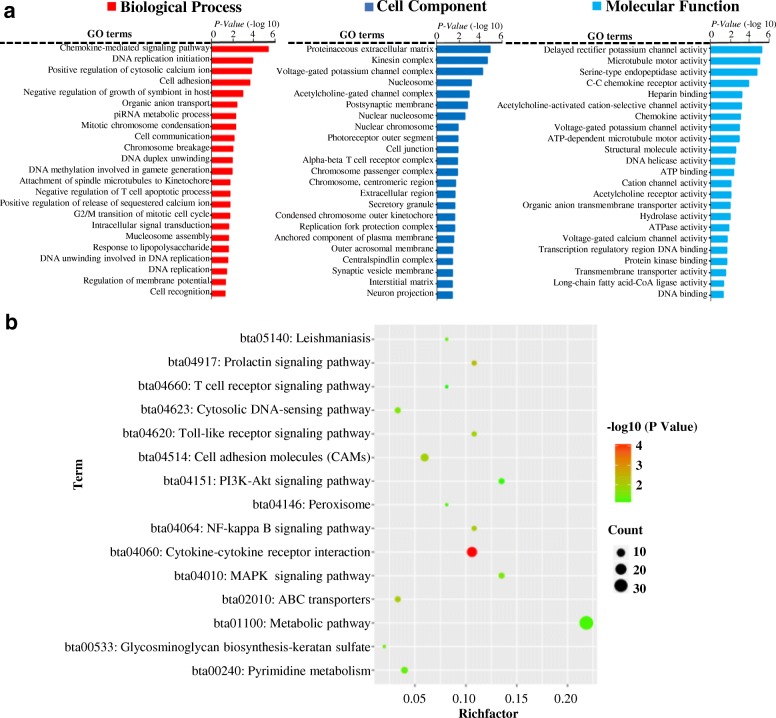
Functional enrichment analysis of lncRNAs target genes in cis and trans. **a** Gene Ontology (GO) analysis of lncRNAs target genes in *cis* and *trans*. The results indicated that those genes seem to play an essential role in lactation-related pathways, including phosphorylation, protein glycosylation, cytokine-mediated, and positive regulation of cell division signaling pathway, etc. **b** KEGG pathway enrichment analysis of lncRNAs target genes in *cis* and *trans*. These KEGG terms were lactation-associated pathways, including NF-kappa B, MAPK, PI3K-Akt, prolactin, Toll-like receptor, and CAMs signaling pathway, etc.

A total of 47 potential target genes *in cis* of lncRNAs (Additional file 10) were selected to carry out functional enrichment analysis. Our results showed that nine significantly enriched GO terms focused on the negative regulation of JAK-STAT cascade (GO: 0046426), positive regulation of transcription from RNA polymerase II promoter (GO: 0045944), cytokine-mediated signaling pathway (GO: 0019221), and positive regulation of phosphorylation (GO: 0042327), etc. (Fig. 7a, *P* < 0.05). Previous study had suggested that JAK-STAT pathway made a marked contribution to dairy milk production traits [49]. In addition, phosphorylation regulation of mammary *S6 K1* and *eIF2* genes could control milk protein yield [50].

Similarly, a total of 459 potential target genes *in trans* of lncRNAs (Additional file 11) were selected to perform functional enrichment analysis (Fig. 7a, *P* < 0.05). These terms were protein glycosylation (GO: 0006486), positive regulation of cell division (GO: 0051781), DNA-directed RNA polymerase III complex (GO: 0005666), and growth factor binding signaling pathway (GO: 0019838), etc. It was worth mentioning that cell division signaling pathway had been demonstrated to be closely associated with mammary gland architecture construction and maintenance [42]. Additionally, protein glycosylation in mammal membrane played important roles in milk quality and biomodulator properties [51].

Meanwhile, the KEGG results from the target genes of candidate lncRNAs *in trans* and *cis* illustrated 15 pathways (Fig. 6b). Among these pathways, PI3K-Akt and MAPK pathways had been confirmed to be associated with cow lactation [43, 52].

### Interaction network of protein-protein

**Fig. 8:**
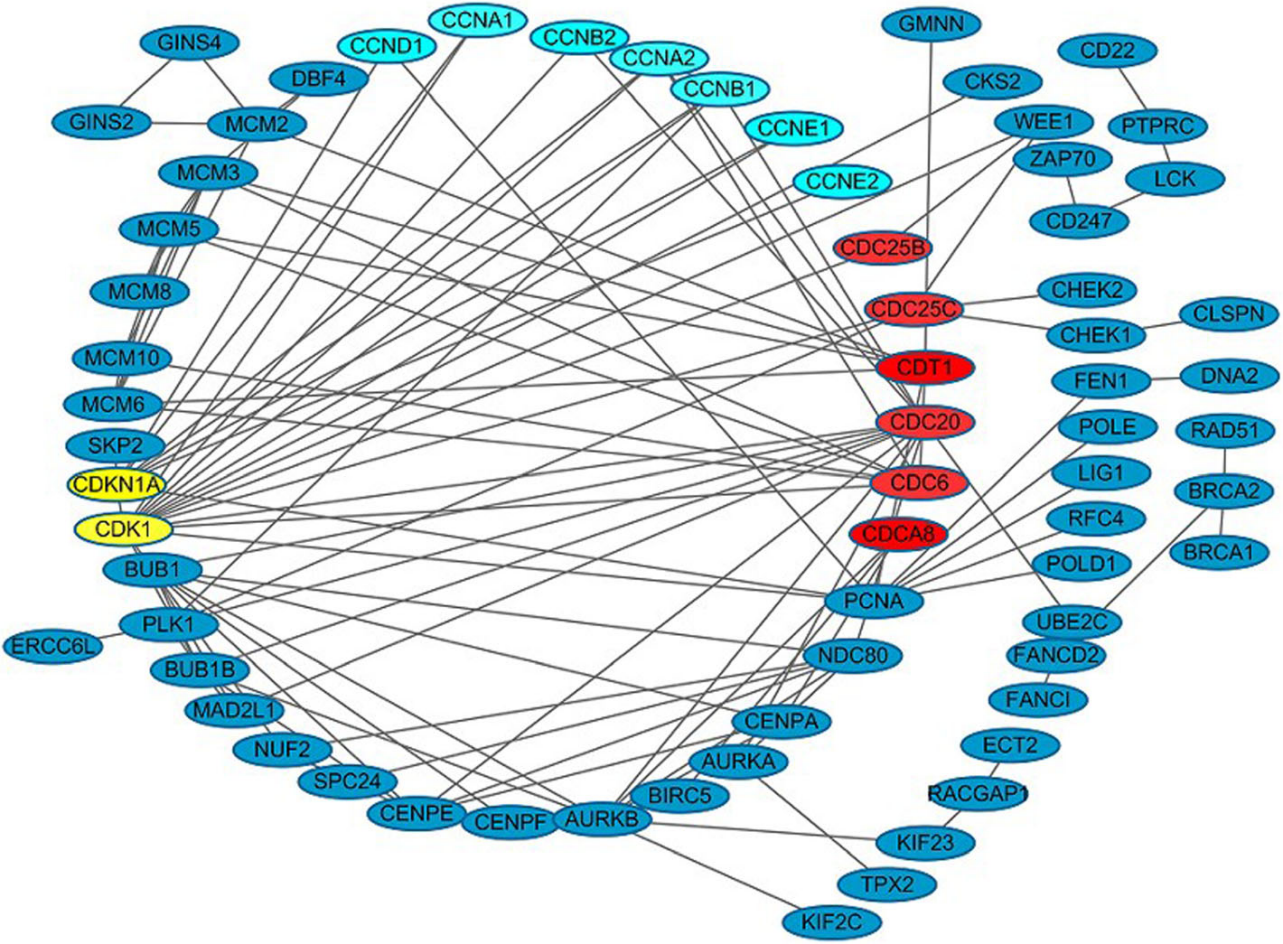
Protein-protein network was analyzed using the String. Cyclin-associated proteins were depicted as light blue frame, cell division cycle-associated protein as red oval symbol, cyclin dependent kinase-associated protein as yellow frame. Their associated proteins were depicted as dark blue

Combine the bioinformatics analysis of DEGs and target genes of DELs, the interaction network between proteins was produced using String software (Fig. 8). The results revealed that protein-protein interaction focused on CCN (CCND1, CCNA1, CCNB2, CCNA2, CCNB1, CCNE1, and CCNE2) and CDC protein family (CDC6, CDC20, CDC25B, CDC25C, and CDCA8). CCN and CDC protein families had been demonstrated to be involved in cyclin and cell division cycle, respectively [53, 54].

### qRT-PCR

**Fig. 9:**
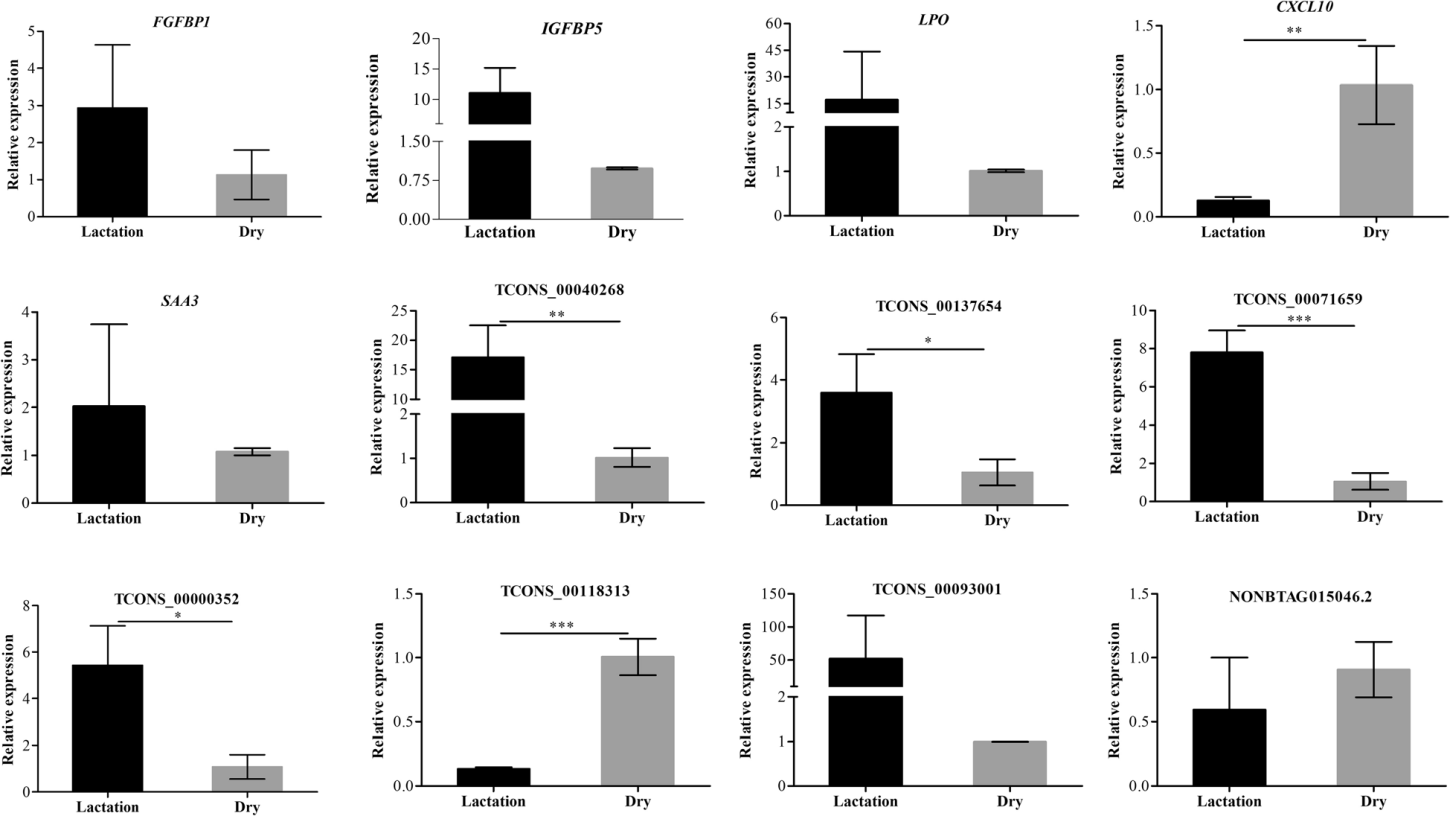
Validation of DEGs and DELs in dry and lactation periods by qRT-PCR. Relative expression of DEGs (*FGFBP1*, *IGFBP5*, *LPO*, *CXCL10*, and *SAA3*) and DELs (TCONS_00040268, TCONS_00137654, TCONS_00071659, TCONS_00000352, TCONS_01118313, TCONS_ 00093001 and NONBTAG0105046.2) were verified by qRT-PCR, and the results indicated that the relative expressions of TCONS_00040268, TCONS_00137654, TCONS_00071659, TCONS_00000352 and TCONS_ 00093001 in lactation were significantly higher than that in dry stage (*P* < 0.05). On the contrary, *CXCL10* and TCONS_01118313 in lactation were lower than that in dry stage (*P* < 0.05). The qRT-PCR results were consistent with the sequencing data

Seven lncRNAs and five genes, which were significantly differentially expressed between the two periods, were selected to verify the transcriptome sequencing data using qRT-PCR. The seven lncRNAs were selected based on maximal fold change (and *P* < 0.05) and location of the genome. For the five genes, they were selected because of their fold change (and *P* < 0.05) and potential function in lactation. The results showed that the relative expression of the selected lncRNAs and genes was consistent with their sequencing data (Fig. 9).

## Discussion

Increasing data had shown that lncRNAs could regulate gene expression both at transcriptional and post-transcriptional levels, including the regulation of splicing, mRNA processing, and translation [55]. It had been demonstrated that some lncRNAs implicated in breast normal development and cancer based on their expression pattern, function and localization in human and mouse [56, 57]. However, limited researches had been reported the regulation mechanism of lncRNAs on bovine lactation. Koufariotis et al. (2015) identified and annotated novel lncRNA transcripts in the bovine genome across 18 tissues (including mammary gland) via RNA sequencing. In addition, they found most lncRNA in bovine mammary gland were downregulated compared with other tissues [58]. Tong et al. (2017) identified 36 lincRNAs located in milk related quantitative trait loci (QTL) from five RNA-seq datasets of bovine mammary glands whereas one lincRNA was within clinical mastitis QTL region, which indicated that these lncRNAs were involved in many biological functions including susceptibility to clinical mastitis as well as milk quality and production [59]. In our study, lactation-related lncRNAs were assessed by directly lncRNA-seq from cow mammary glands at the dry and lactation period, respectively. Given the functional enrichment analysis of lncRNAs target genes, 24 lncRNAs were identified to have potential role in lactation (Table 3). Previous study had suggested that lncRNAs could regulate biological processes by sponging miRNAs [28], so 15 lncRNAs interacted with lactation-associated miRNAs were also considered as potential regulators for lactation (Table 2). Among those lncRNAs, five lncRNAs were shared by the above two prediction methods.

### LncRNAs might regulate expression of lactation-associated genes in trans or cis

It was well recognized that a series of genes involved in lactation initiation, maintenance as well as mammary gland growth, development and breast cancer by direct or indirect regulation [8, 9, 11, 60]. In this study, the DEGs functional enrichment results showed that these genes were related to some biological processes, including cell division, adhesion, cycle, ERK1 and ERK2 cascade, PI3K-Akt, PPAR, and TNF pathway, which were closely associated with lactation [43–46].

LncRNAs can regulate neighboring gene expression, therefore high expression correlations exert between lncRNAs and mRNAs (known as in *cis*) [61]. LncRNAs also can change the expression of distant mRNAs through the pairing of lncRNAs-mRNA (known as in *trans*). Bioinformatics analysis of lncRNAs target genes in *trans* showed that these genes played an important role in some pathways, such as MAPK, PI3K-Akt, prolactin, NF-kappa B, and Toll-like receptor signaling pathways, which played important roles during mammary gland development and lactation [4, 8, 62]. Consequently, many lncRNAs might function through targeting mRNA which played important roles in mammary gland from non-lactation to lactation period. For example, lncRNA TCONS_00075230 and *IL1B* had an opposite expression trend between dry and lactation period, and *IL1B* had reported to inhibit the proliferation of normal human mammary epithelial cells function as a retinoic acid induced gene [48]. So TCONS_00075230 might involve in lactation process through altering the expression of *IL1B*.

### Regulating role of lncRNA-miRNA-mRNA network

It was known that the regulatory networks of lncRNAs, miRNA, and ceRNAs communicated with each other to regulate gene expression [28]. LncRNA *SNHG7* could accelerate prostate cancer proliferation and cell cycle progression through *cyclin D1* by sponging miR-503 [63]. The axis of lncRNA *H19*-miR-675-*TGFBI* had possible diagnostic and therapeutic potential for advanced prostate cancer [64]. LncRNA *APF* could control the expression of miR-188-3p, which suppressed myocardial infarction and autophagy by targeting *ATG7* [65]. A series of miRNAs had been demonstrated to regulate lactation-associated processes, including lactation cycle, milk fat accumulation, hormone receptor activity, mammary gland involution and development, and lactation activity of mammary epithelial cells [14, 66, 67]. In this study, some candidate lncRNAs, including TCONS_00040268, TCONS_00137654, TCONS_00071659, and TCONS_00000352, could interact with lactation-related miR-221, and miR-221 could target *ErBb3* gene. It had known that *ErBb3* gene could drive mammary epithelial survival and differentiation during pregnancy and lactation [11]. Therefore, these lncRNAs might play an important role in regulating lactation, which would provide a new insight into lactation process of cattle.

## Conclusion

In this study, 3746 significantly dysregulated lncRNA transcripts were found in lactation period compared with that in dry period, including 2732 down- and 1014 up-regulated lncRNA transcripts (fold change≥2, *P* < 0.05). Functional enrichment analysis of target genes and interacted miRNAs prediction of 34 lncRNAs indicated these lncRNAs might be important regulators for lactation in dairy cattle. This study would provide a resource for bovine lncRNA study involving in lactation biology.

## Additional files

Additional file 1: qRT-PCR primers for lncRNAs and coding genes. (DOCX 21 kb)

Additional file 2: The expression profile of DEGs in the dry and lactation periods. (XLSX 387 kb)

Additional file 3: The expression profile of novel lncRNAs in the dry and lactation periods. (XLSX 980 kb)

Additional file 4: The sequence information of novel lncRNAs. (XLSX 2011 kb)

Additional file 5: The expression profile of annotated lncRNAs in the dry and lactation periods. (XLSX 1732 kb)

Additional file 6: The expression file of novel DELs in the dry and lactation periods. (XLSX 230 kb)

Additional file 7: The expression file of annotated DELs in the dry and lactation periods. (XLSX 224 kb)

Additional file 8: Stage specific expressed lncRNAs in the dry or lactation periods. (XLSX 41 kb)

Additional file 9: Stage specific expressed genes in the dry or lactation periods. (XLSX 21 kb)

Additional file 10: Predicted target genes and mRNAs of DELs (in *cis*). (XLSX 24 kb)

Additional file 11: Predicted target genes and mRNAs of DELs (in *trans*). (XLSX 84 kb)

## Abbreviations

BMECs: Bovine mammary epithelial cells
ceRNAs: Competing endogenous RNAs
DEGs: Differentially expressed genes
DELs: Differentially expressed lncRNAs
DMEM: Dubecco’s modified Eagle medium
lncRNAs: Long non-coding RNAs
miRNAs: MicroRNAs
qRT-PCR: Quantitative real time PCR
