# Supplementary material for: Transcriptome sequencing to detect the potential role of long non-coding RNAs in bovine mammary gland during the dry and lactation period
Source: BMC Genomics. 2018 Aug 13;19:605. doi: 10.1186/s12864-018-4974-5 (PMC6090732; doi:10.1186/s12864-018-4974-5)
Supplement: Supplementary file 1 — qRT-PCR primers for lncRNAs and coding genes. (DOCX 21 kb) [file 12864_2018_4974_MOESM1_ESM.docx]

**Additional file 1**

Supplemental Table 1. qRT-PCR primers for lncRNAs and coding genes

| Genes name | Forward and reverse primer (5'→3') | Product length (bp) |
| --- | --- | --- |
| TCONS_01118313 | F: TTGCTCAGGTGATAGTGATT | 197 |
|  | R: GAAGGTTGTCCAAGGTATATG |  |
| TCONS_00093001 | F: GTTGGCTCCTGAACCTTG | 168 |
|  | R: ATCGTACTGCTTGAGTGTAG |  |
| TCONS-00040268 | F: AAGTGTGTGCAAATGGGTTAG | 146 |
|  | GAAACAGAGGCTCAGAGACAG |  |
| TCONS-00071659 | F: TATTGAGTGACTTTCATCGTA | 192 |
|  | R: TAGAGTTTGAGATTTCTTGGA |  |
| TCONS-00137654 | F: GCAGTGACATAGAGTTACATT | 218 |
|  | R: ATTGTTCTTTCTCTTCCTTTC |  |
| TCONS-00000352 | F: TCTGTGTCCCTTCTTACTGTC | 164 |
|  | R: CATTCATCAAAACCCATAGAG |  |
| NONBTAG015046.2 | F: GGATCAGACTACTTCGCAAA | 171 |
|  | R: CTCTCACATTATGGAACACATG |  |
| *ERBb3* | F: TGAACTACAACACCAACTCTA | 168 |
|  | R: TCACTACTATCTCAGCATCTC |  |
| *FGFBP1* | F: GACCTGCACGCTCAGAACTA | 136 |
|  | R: CCACGTCTATTCCTTCCTTCCT |  |
| *IGFBP5* | F: CAACTGTGACCGCAAAGGG | 150 |
|  | R: TCGAAGGTGTGGCACTGAA |  |
| *SAA3* | F: CCTTTCCACGGGCATCATTT | 123 |
|  | R: TTCATGTCTTGGTAAGCTCTCC |  |
| *CXCL10* | F: GCAATAAGGAATGGACGCTGTT | 159 |
|  | R: TGGGTTTAGGCAGGCTTCAT |  |
| *LPO* | F: CTTACAGCGTTGCCGAGAC | 177 |
|  | R: CCTCCAATCCAGATGTCAATGT |  |
| *GAPDH* | F: GATGGTGAAGGTCGGAGTGA | 178 |
|  | R: CTTGACTGTGCCGTTGAACT |  |
